# Improving heterologous protein production by modulating ROS homeostasis in *Nicotiana benthamiana*

**DOI:** 10.1186/s43897-026-00260-9

**Published:** 2026-08-02

**Authors:** Yuanxin Wu, Qi Ni, Sayed Abdul Akher, Zenglin Zhang, Jie Wang, Yongfeng Guo, Jianfeng Zhang

**Affiliations:** 1 Tobacco Research Institute, Chinese Academy of Agricultural Sciences, Qingdao, China; 2 Beijing Life Science Academy (BLSA), Beijing, China; 3 Key Laboratory of Biosynthesis and Biomanufacturing in Model Plants (Beijing Life Science Academy), Ministry of Industry and Information Technology, Beijing, China

*Agrobacterium tumefaciens*–mediated transient expression systems have emerged as versatile platforms for the rapid production of recombinant proteins in plants, widely used in molecular farming and plant biotechnology (Buyel, 2018; Moon, 2019). Owing to their low risk of pathogenic contamination and inherent scalability, these systems enable high-level expression within short timeframes, making them particularly attractive for manufacturing antibodies, vaccines, and other therapeutic proteins (Nosaki, 2021). However, certain recombinant proteins, especially those derived from animal or microbial sources, can trigger strong stress responses or exhibit cytotoxicity in plant hosts, resulting in reduced protein yields (Liu and Timko, 2022; Shi, 2019). Excessive accumulation of reactive oxygen species (ROS) is a major contributor to such stress, disrupting cellular homeostasis, inducing leaf necrosis, and ultimately limiting protein production in host plants such as *Nicotiana benthamiana* (Nosaki, 2021). Despite this, broadly applicable strategies to mitigate ROS-associated damage remain underexplored.

Human phospholysine phosphohistidine inorganic pyrophosphate phosphatase (hLHPP) has recently been identified as a tumor suppressor involved in regulating cell growth and polarity (Hindupur, 2018; Wu, 2022). Its downregulation is associated with multiple malignancies, including renal, liver, and colorectal cancers (Hou, 2020; Wu, 2022). LHPP-based therapeutic strategies have shown promising anti-proliferative effects (Zhao, 2024). However, plant-based production of hLHPP has not yet been established.

When recombinant hLHPP (rhLHPP) was transiently expressed in *N. benthamiana* leaves using the pJL-TRBO vector, severe necrosis developed progressively from 5 days post-infiltration (dpi) (Fig. 1A). Immunoblot analysis detected rhLHPP accumulation from 1 to 6 dpi, peaking at 5 dpi, with an estimated yield of 54.23 mg kg⁻^1^ fresh weight at 6 dpi (Fig. 1B, Fig. S1). In contrast, expression of GFP using the same vector produced only mild chlorosis (Fig. 1A, Fig. S1E). Comparable results were obtained using the pEAQ-*HT* system, where rhLHPP—but not GFP—induced necrosis (Fig. S2). These findings indicate that the observed phenotype is specifically associated with rhLHPP accumulation rather than vector-related effects.

**Fig. 1:**
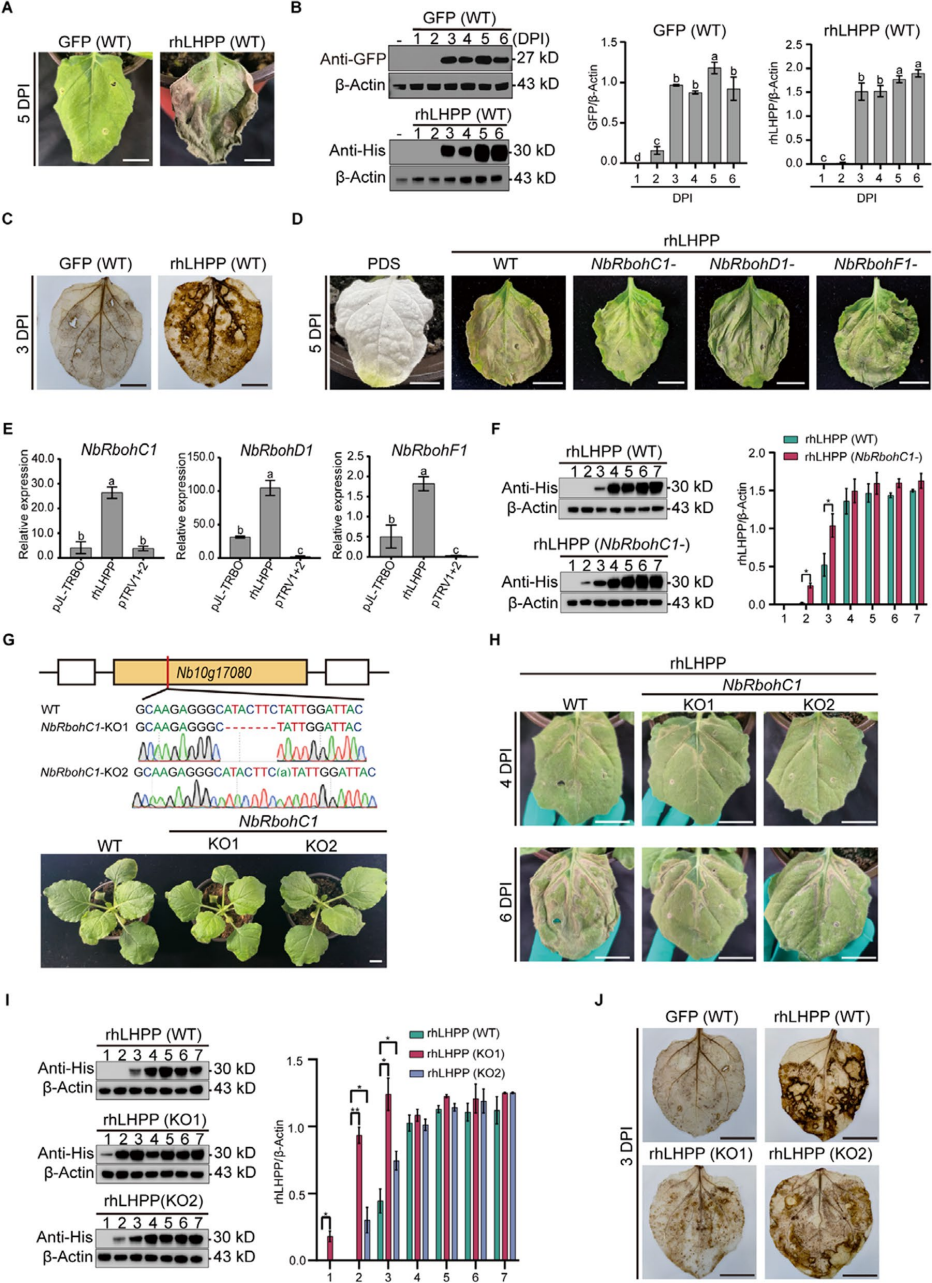
Knockout of *NbRbohC1* alleviates rhLHPP-induced leaf necrosis and enhances recombinant protein accumulation in *Nicotiana benthamiana.***A** Phenotypes of leaves expressing pJL-TRBO-rhLHPP or pJL-TRBO-GFP at 5 dpi. **B** Western blot and relative quantification of rhLHPP (~30 kDa) and GFP (~27 kDa) from 1–6 dpi. β-Actin (~43 kDa) was used as a loading control. Band intensities were quantified relative to β-Actin. Different letters indicate statistically significant differences. “-”, extract from leaves injected with empty vector. **C** DAB staining of *N. benthamiana* leaves transiently expressing GFP or rhLHPP at 3 dpi. **D**, **E** Leaf phenotypes and relative *NbRboh* expression levels following VIGS silencing. PDS, VIGS bleached control; rhLHPP, indicates hLHPP expression in wild-type *N. benthamiana*; pTRV1 + 2, represents hLHPP expression in *N. benthamiana* treated with pTRV1 + pTRV2. Different letters indicate statistically significant differences. **F** Western blot analysis of rhLHPP expression in WT and *NbRboh*-silenced leaves. Specific bands at ~30 kDa (rhLHPP) and ~43 kDa (β-Actin, loading control) are shown, with quantification relative to β-Actin. **G** Schematic diagram of *NbRbohC1* mutation sites and growth phenotypes of *NbRbohC1* KO1 and KO2 mutant plants under normal conditions. **H** Leaf phenotype with hLHPP transiently expressed in WT and the *NbRbohC1* mutant leaves. **I** Protein detection of hLHPP transiently expressed in WT and the *NbRbohC1* mutant leaves. Grayscale comparison of transiently expressed hLHPP protein. **J** DAB staining of hLHPP transiently expressed in in WT and the *NbRbohC1* mutant leaves. Data were analyzed using one-way ANOVA followed by Tukey’s multiple-comparison test (*n* = 3); *, 0.01 ≤ *p* < 0.05; **, 0.001 ≤ *p* < 0.01; scale bar = 2 cm

Subcellular targeting is known to enhance recombinant protein stability and mitigate cytotoxicity (Liu and Timko, 2022; Shi, 2019; Nosaki, 2021). To assess this, rhLHPP was directed to the endoplasmic reticulum (ER), apoplast, or vacuole using established targeting signals (Fig. S3A–C). Co-localization with compartment-specific markers confirmed proper targeting (Fig. S3B). Among these, ER-localized rhLHPP exhibited the highest accumulation and minimal necrosis, with sustained expression peaking at 6 dpi (Fig. S3D–F). In contrast, apoplast-targeted rhLHPP showed early but transient accumulation, while vacuolar targeting led to low protein levels, likely due to degradation. These data suggest that the ER provides a favorable environment for rhLHPP stability and alleviates its cytotoxic effects.

Given the reduced toxicity observed with ER targeting, we next investigated whether ROS accumulation underlies rhLHPP-induced necrosis. DAB and NBT staining revealed significant accumulation of hydrogen peroxide and superoxide anions at 3 dpi in rhLHPP-expressing leaves (Fig. 1C; Fig. S5A). ER targeting markedly reduced ROS levels, supporting a link between ROS overproduction and cell death. Treatments with ROS scavengers (ascorbic acid and acetylsalicylic acid) significantly alleviated both ROS accumulation and necrosis (Fig. S4). Similarly, co-expression of ROS-scavenging enzymes (NbCAT2, NbSOD1, NbGPX1) reduced oxidative stress and improved leaf viability (Fig. S5B–F). Together, these results demonstrate a causal role of ROS in rhLHPP-induced damage.

In plants, NADPH oxidases (Rbohs) are major sources of ROS (Angelos and Brandizzi, 2018; Møller, 2007; Yu, 2020). Expression profiling identified NbRbohC1, NbRbohD1, and NbRbohF1 as being strongly induced during rhLHPP expression (Fig. S6A, B). Virus-induced gene silencing revealed that suppression of *NbRbohC1* most effectively attenuated leaf necrosis, whereas silencing *NbRbohD1* or *NbRbohF1* had minimal effects (Fig. 1D, E). Correspondingly, rhLHPP accumulation was higher and detectable earlier in *NbRbohC1*-silenced plants (Fig. 1F; Fig. S6C, D), suggesting that NbRbohC1 is the primary driver of ROS-mediated toxicity.

To further validate this finding, we generated *NbRbohC1* knockout lines using CRISPR/Cas9. These mutants exhibited normal growth under standard conditions (Fig. 1G). Upon rhLHPP expression, the knockout lines showed markedly reduced necrosis, decreased ROS accumulation, and enhanced protein expression in comparison with WT (Fig. 1H–J). These results confirm that suppression of *NbRbohC1* significantly improves rhLHPP production efficiency in *N. benthamiana*.

In summary, our study identifies oxidative stress as a key bottleneck limiting efficient recombinant hLHPP production in plants. Cytoplasmic expression of rhLHPP triggers excessive ROS accumulation, leading to cell death and reduced yield. Strategies such as ER targeting, ROS scavenging, and disruption of *NbRbohC1* effectively mitigate these effects. Notably, *NbRbohC1* knockout plants represent a promising chassis for improving production of rhLHPP and potentially other cytotoxic recombinant proteins. While the broader applicability of this approach requires further validation, systematic identification and engineering of ROS-producing pathways may provide a general strategy to overcome phytotoxicity in plant molecular farming. Future work may focus on combining ROS suppression with additional modifications—such as reduced protease activity or inducible expression systems—to create optimized platforms for large-scale biomanufacturing.

## Supplementary Information

Supplementary Material 1.

Supplementary Material 2.

Supplementary Material 3.

## Data Availability

The datasets used and/or analysed during the current study are available from the corresponding author on reasonable request.
