# Supplementary material for: Improving heterologous protein production by modulating ROS homeostasis in Nicotiana benthamiana
Source: Mol Hortic. 2026 Aug 2;6:62. doi: 10.1186/s43897-026-00260-9 (PMC13429016; doi:10.1186/s43897-026-00260-9)
Supplement: Supplementary file 1 — Supplementary Material 1. [file 43897_2026_260_MOESM1_ESM.docx]

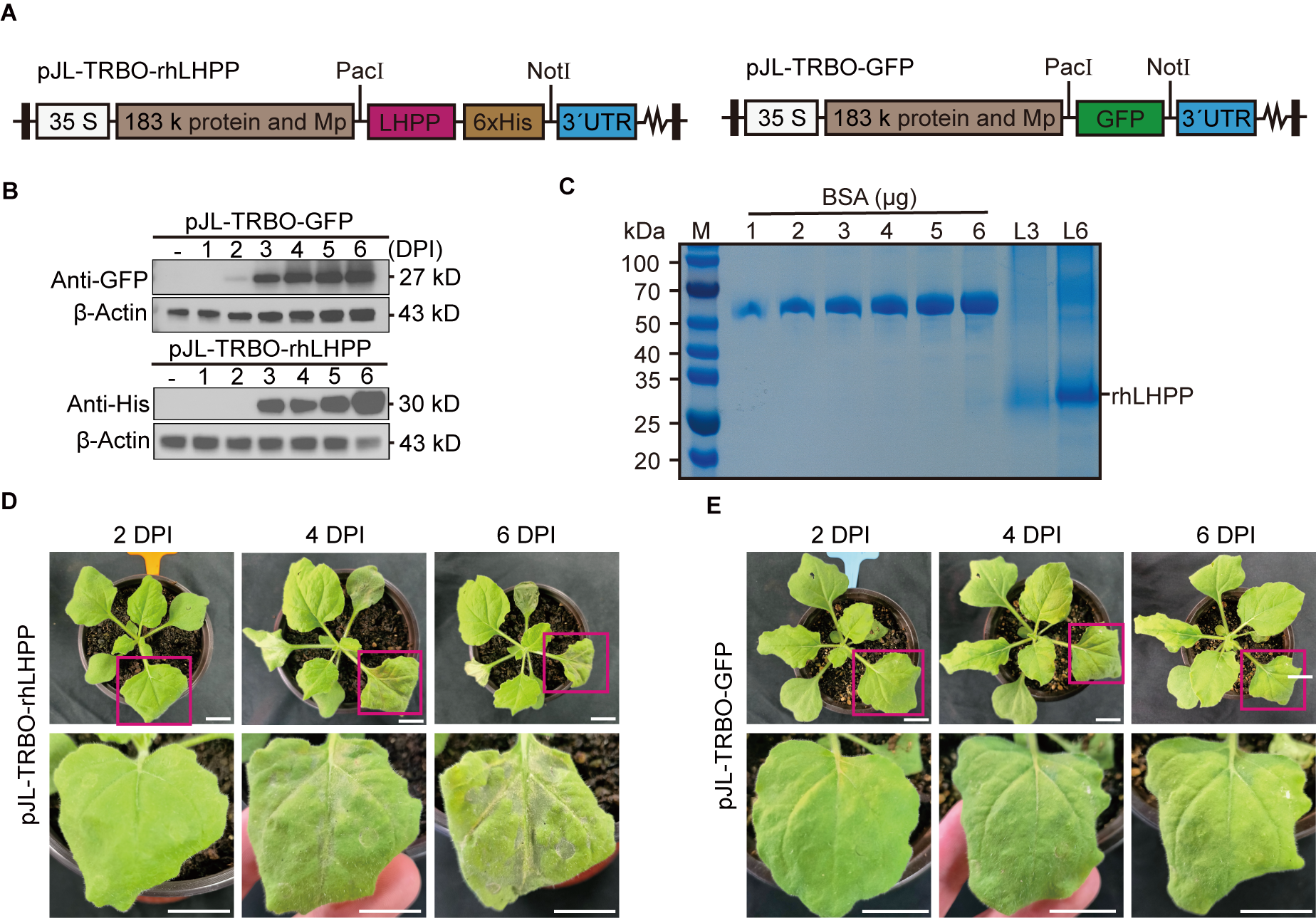


**Fig S1. Transient expression of rhLHPP with pJL-TRBO vector leads to leaf necrosis in *N. benthamiana*.**

(A) Schematic diagrams of rhLHPP and GFP constructs inserted into pJL-TRBO via *Pac*Ⅰ and *Not*Ⅰ.

(B) Western blot analysis of rhLHPP (~30 kDa) and GFP (~27 kDa) expression from 1–6 dpi, with β-Actin (~43 kDa) as a loading control. “-”, extract from leaves injected with empty vector.

(C) SDS-PAGE and Coomassieblue staining of rhLHPP purified from *N. benthamiana* via Ni-NTA affinity chromatography. 1-6 : represent the mass (μg) of BSA standard proteins; L3, purified extract from pJL-TRBO-rhLHPP-infiltrated *N. benthamiana*, 3 dpi; L6, purified extract from pJL-TRBO-rhLHPP-infiltrated *N. benthamiana*, 6 dpi.

(D, E) Phenotypic changes of leaves infiltrated with *Agrobacterium* carrying pJL-TRBO-rhLHPP or pJL-TRBO-GFP at 2, 4, and 6 dpi. Enlarged boxed areas are shown below; scale bar = 2 cm.


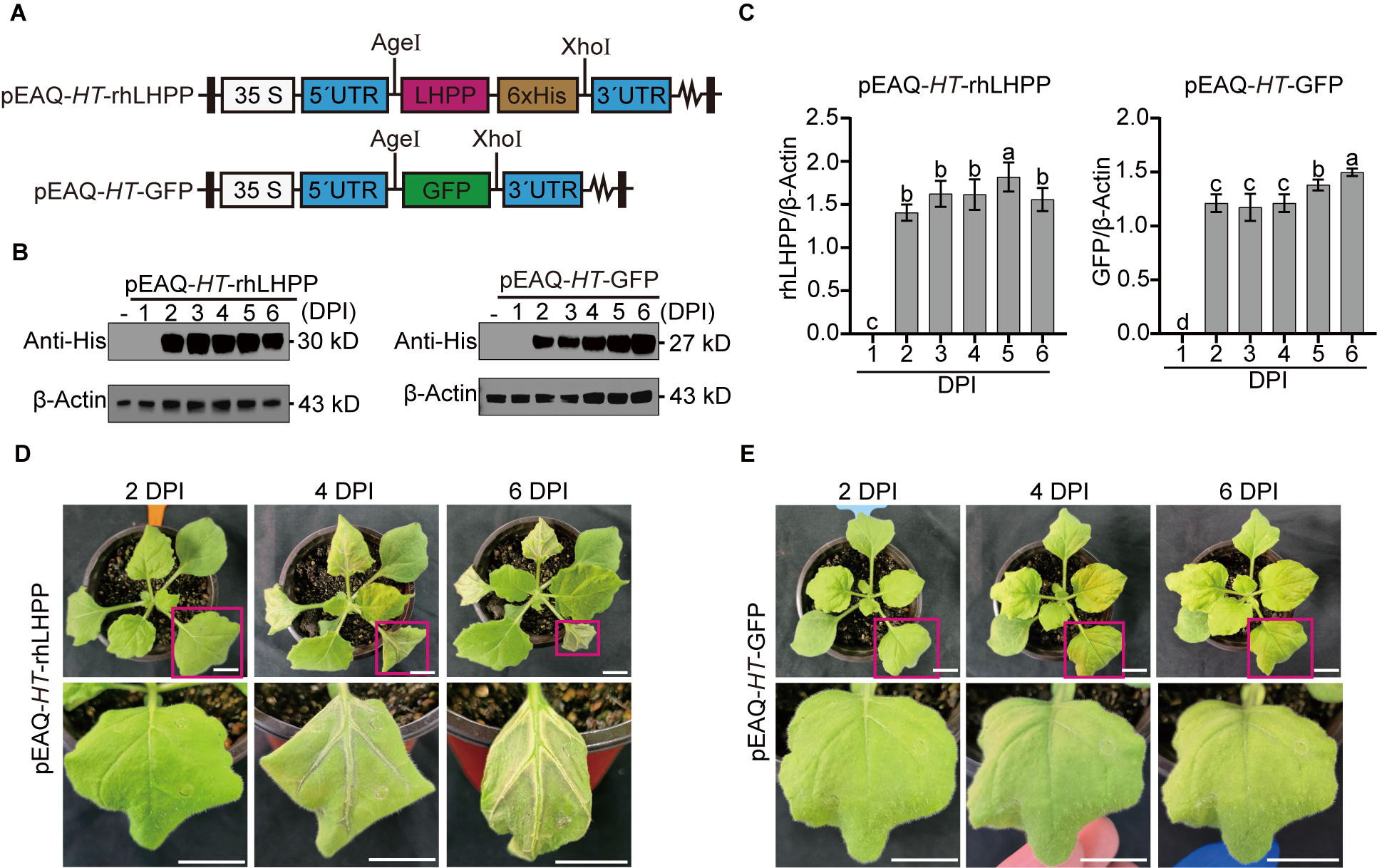


**Fig S2. Transient expression of rhLHPP with the pEAQ-*HT* vector causes leaf necrosis in *N. benthamiana*.**

(A) Schematic diagrams of rhLHPP and GFP constructs in the pEAQ-*HT* vector, inserted via *Age*Ⅰ and *Xho*Ⅰ sites.

(B) Western blot detection of rhLHPP (~30 kDa), GFP (~27 kDa), and β-Actin (~43 kDa, loading control) from 1–6 dpi. “-”, extract from leaves injected with empty vector.

(C) Quantification of protein expression relative to β-Actin. Different letters indicate statistically significant differences.

(D, E) Phenotypic changes of leaves infiltrated with *Agrobacterium* carrying pEAQ-*HT*-rhLHPP or pEAQ-*HT*-GFP at 2, 4, and 6 dpi. Enlarged views of boxed areas are shown below; scale bar = 2 cm.

Data were analyzed using one-way ANOVA followed by Tukey’s multiple-comparison test (n=3).


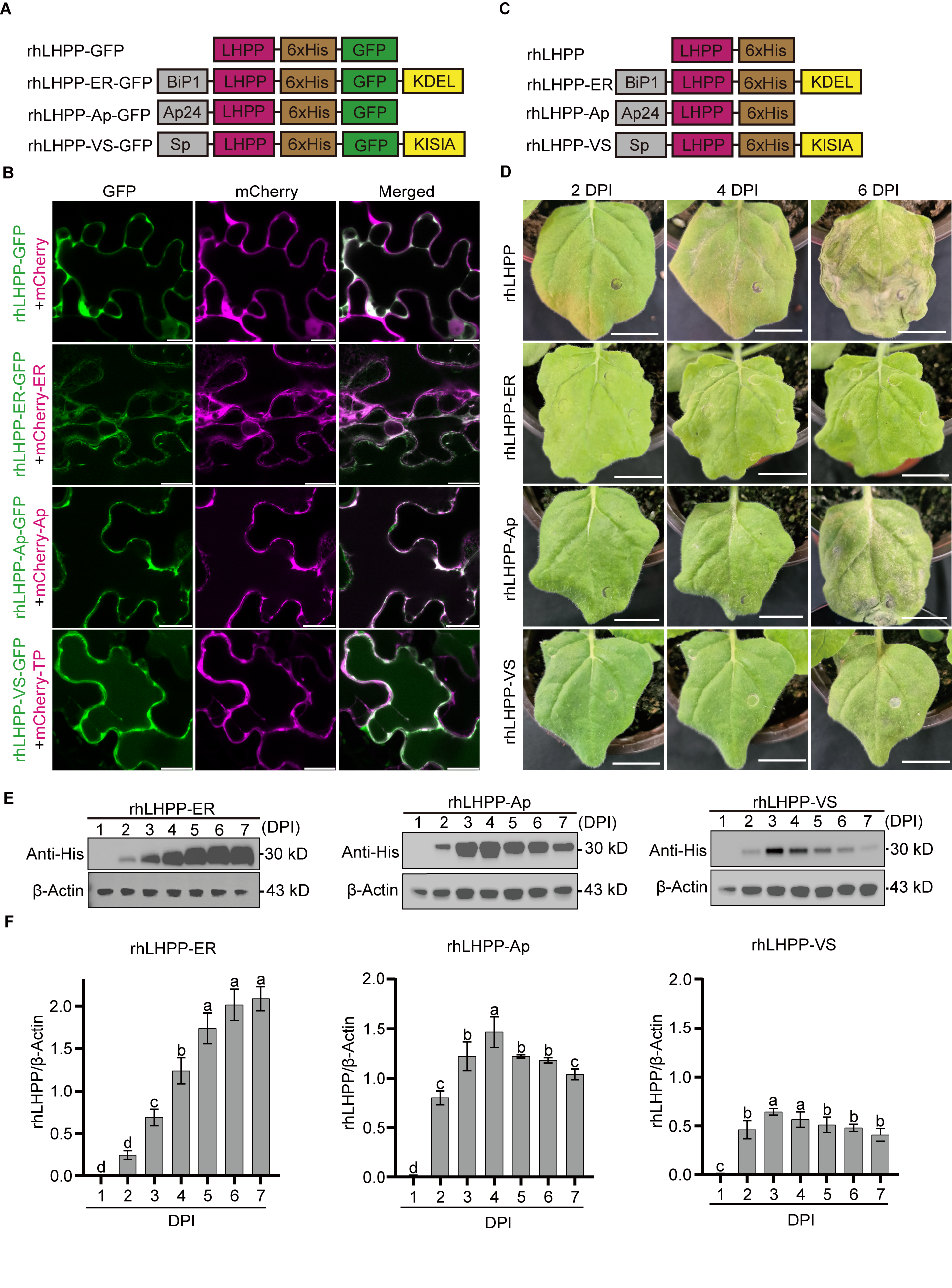


**Figure S3. The effects of compartmentalized expression of rhLHPP on leaf phenotypes and protein accumulation.**

(A) Schematic diagram of rhLHPP localization constructs. N-terminal signal peptides BiP1 or Sp were used for ER targeting, with C-terminal ER-retention (KDEL) or vacuole-retention (KISIA) signals, respectively. An apoplast-targeting signal (Ap24) was added alone to the N-terminus for secretion.

(B) Subcellular localization of rhLHPP proteins under different signal peptides observed by confocal microscopy. Co-localization was performed with differently targeted mCherry markers; scale bar = 20 μm.

(C) Schematic diagram of rhLHPP compartmentalized expression vectors without the GFP sequence at the 3' end.

(D) Phenotypes of leaves infiltrated with *Agrobacterium* expressing rhLHPP, rhLHPP-ER, rhLHPP-Ap, and rhLHPP-VS at 2, 4, and 6 dpi; scale bar = 2 cm.

(E, F) Western blot analysis of rhLHPP with different targeting signals and quantification relative to β-Actin (~43 kDa, loading control) respectively. Specific bands for rhLHPP (~30 kDa) were observed. Different letters indicate statistically significant differences.

Data were analyzed using one-way ANOVA followed by Tukey’s multiple-comparison test (n=3).


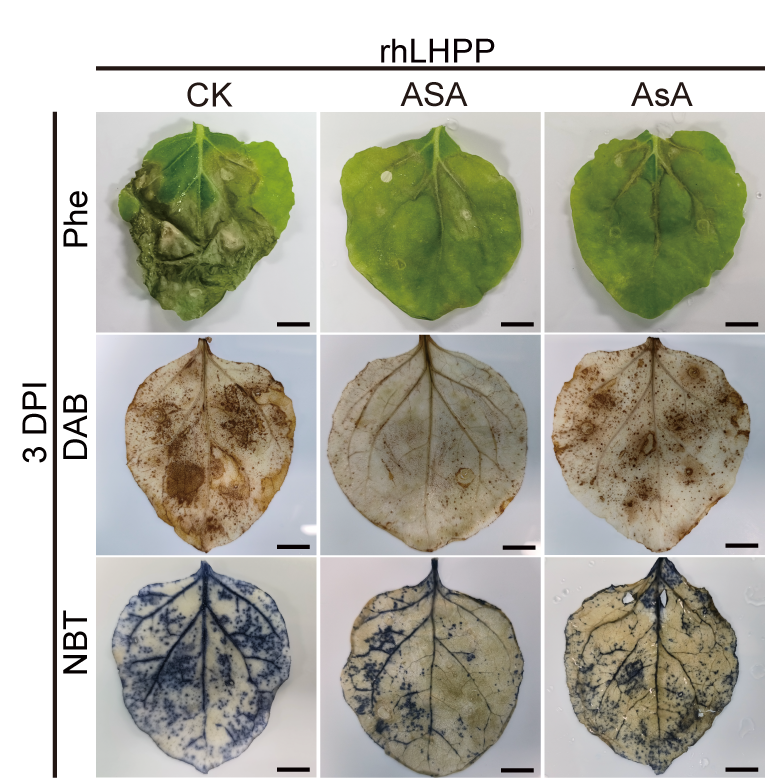


**Fig S4: Exogenous acetylsalicylic acid (ASA) and ascorbic acid (AsA) modulate *N. benthamiana* leaf phenotypes and ROS accumulation.**

During *Agrobacterium*-mediated transient expression, *N. benthamiana* leaves were sprayed with 1 mg /mL ASA or AsA immediately after infiltration and re-treated daily. By the third day, phenotype and ROS deposition were assessed via DAB and NBT staining. rhLHPP, leaves infiltrated with *Agrobacterium* harboring pJL-TRBO-rhLHPP; CK: ddH₂O spray; AsA: ascorbic acid spray; ASA: acetylsalicylic acid spray; scale bar = 1 cm.


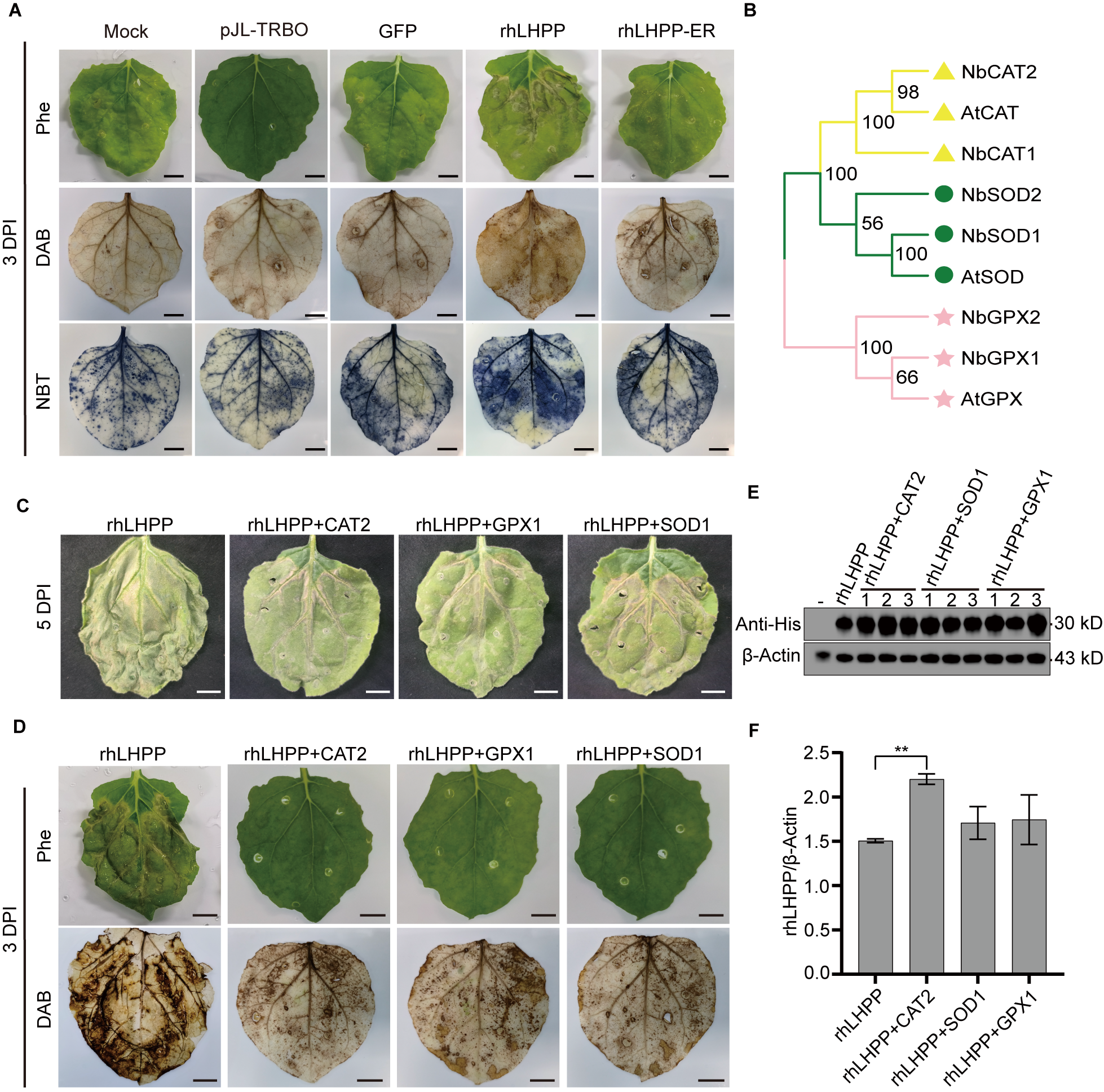


**Fig S5. ROS accumulation and protein yield of rhLHPP when co-expressed with ROS-scavenging enzymes.**

(A) DAB and NBT staining of *N. benthamiana* leaves transiently expressing GFP or rhLHPP at 3 dpi.

(B) Phylogenetic analysis of catalase, superoxide dismutase, and glutathione peroxidase from *N. benthamiana* and *Arabidopsis*. Numbers indicate correlation values.

(C, D) Phenotypes and DAB staining of tobacco leaves co-infiltrated with rhLHPP and ROS-scavenging enzymes respectively.

(E, F) Western blot analysis of rhLHPP expression in tobacco leaves co-infiltrated with ROS-scavenging enzymes at 5 dpi. Specific bands at ~30 kDa (rhLHPP) and ~43 kDa (β-Actin, loading control) were observed, with quantification relative to β-Actin shown in (F). “-”, extract from leaves injected with empty vector; 1–3, three biological replicates; dpi, days post-infection; scale bar = 1 cm.

Data were analyzed using one-way ANOVA followed by Tukey’s multiple-comparison test (n=3); **, 0.001 ≤ p < 0.01


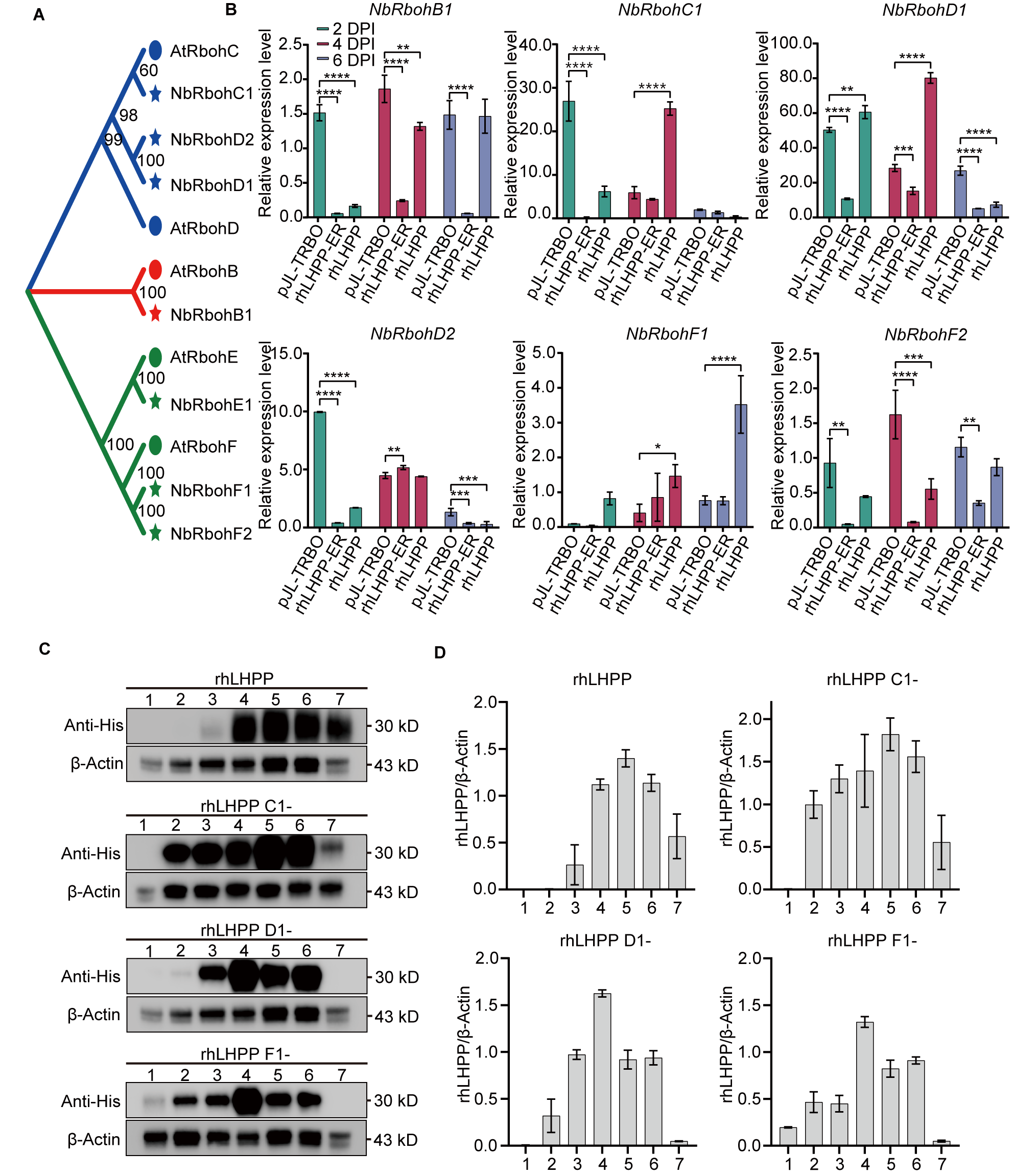


**Fig S6. Effects of VIGS-silencing *NbRbohs* on rhLHPP expression and leaf phenotypes.**

(A) Phylogenetic analysis of *Rboh* proteins from *N. benthamiana* (7) and *Arabidopsis thaliana* (5). A maximum likelihood tree was constructed using MEGA 11.0; stars denote *N. benthamiana* and circles denote *Arabidopsis*. Numbers indicate correlation values.

(B) RT-qPCR validation of six selected *NbRboh* genes. Relative expression levels are shown compared to β-Actin.

(C,) Western blot analysis was conducted to assess protein expression levels from 1 to 7 dpi.

(D)Relative abundance of rhLHPP was quantified by densitometric analysis with Image J software and normalized to the corresponding β-actin signal. rhLHPP, represents the injection of pJL-TRBO-rhLHPP into *N. benthamiana* leaves that had been pretreated with pTRV1 together with the empty pTRV2 vector; rhLHPP F1−, represents the injection of pJL-TRBO-rhLHPP into *N. benthamiana* after silencing *NbRbohF1*; rhLHPP D1−, represents the injection of pJL-TRBO-rhLHPP into *N. benthamiana* after silencing *NbRbohD1*; rhLHPP C1−, represents the injection of pJL-TRBO-rhLHPP into *N. benthamiana* after silencing *NbRbohC1*.

Statistical analysis was performed by one-way ANOVA.
