# Supplementary material for: Improving heterologous protein production by modulating ROS homeostasis in Nicotiana benthamiana
Source: Mol Hortic. 2026 Aug 2;6:62. doi: 10.1186/s43897-026-00260-9 (PMC13429016; doi:10.1186/s43897-026-00260-9)
Supplement: Supplementary file 2 — Supplementary Material 2. [file 43897_2026_260_MOESM2_ESM.docx]

**Improving heterologous protein production by modulating ROS homeostasis in** ***Nicotiana benthamiana***

**Yuanxin Wu^1,2,3#^, Qi Ni^1#^, Sayed Abdul Akher^1^, Zenglin Zhang^1^, Jie Wang^1,2,3^*, Yongfeng Guo^1^*, Jianfeng Zhang^2,3^***

^1^Tobacco Research Institute, Chinese Academy of Agricultural Sciences, Qingdao, China

^2^Beijing Life Science Academy (BLSA), Beijing, China

^3^Key Laboratory of Biosynthesis and Biomanufacturing in Model Plants (Beijing Life Science Academy), Ministry of Industry and Information Technology, Beijing, China

*Correspondence: Jie Wang ([wangjie06@caas.cn](mailto:wangjie06@caas.cn)); Yongfeng Guo ([guoyongfeng@caas.cn](mailto:guoyongfeng@caas.cn)); Jianfeng Zhang (zhangjf@blsa.com.cn)

**Materials and Methods**

**Plant Materials and Growth Conditions**

*N. benthamiana* plants were grown in a controlled environment chamber under long-day conditions (16 h light/8 h dark) at 24°C. Plants approximately 6-8 weeks old were used for agroinfiltration experiments. The KO1 and KO2 *NbRbohC1* mutant were obtained by CRISPR, the primers information of sgRNA were listed in Table S1.

**Plasmid construction**

Information of primers, plasmids and gene accession numbers are listed in Tables S1-S3 respectively. The coding sequence of human Phospholysine phosphohistidine inorganic pyrophosphate phosphatase protein was obtained from the National Center for Biotechnology Information (NCBI). The sequence was subsequently codon-optimized for *N. benthamiana* and chemically synthesized (GenScript Biotech Corporation, China).

To express recombinant 6×His-rhLHPP, the synthetic gene was inserted into two vectors: pJL-TRBO (*Pac*Ⅰ*/Not*Ⅰ) and pEAQ-*HT* (*Age*Ⅰ*/Xho*Ⅰ). GFP controls were prepared the same way. For subcellular targeting, BiP1-KDEL sent rhLHPP to the ER; Sp-KISIA to the vacuole; Ap24 alone secreted it to the apoplast. All variants were built in pJL-TRBO; GFP fusions were made for imaging. To test if lower ROS boosts yield, *NbCAT2*, *NbSOD1* and *NbGPX1* were cloned into pEAQ-*HT* (*Age*Ⅰ/*Xho*Ⅰ). For VIGS, fragment of Rboh nucleotide sequences were inserted between the *Pst*Ⅰ sites of pTRV2 (pYY13 backbone); pTRV1 and pTRV2-PDS were obtained from stock.

**Transient Expression of recombinant proteins in *N. benthamiana***

After overnight culture, *Agrobacterium tumefaciens* were harvested by centrifugation and resuspended in infiltration buffer [10mM (2-N-morpholino-ethanesulfonic acid (MES), 150 μM acetosyringone and 10mM MgCl_2_, pH 5.6] to obtain a final optical density (OD_600_) of 0.8. The suspensions were then incubated in the dark at room temperature for 2-3 hours. The *Agrobacterium* suspensions was from the abaxial side of leaves from *N. benthamiana* by syringe infiltration and incubated at a dark room for 16 h of cultivation, after which they were moved to a 26 °C greenhouse with a 16 h light/8 h dark cycle for further cultivation. The agroinfiltrated leaves were harvested at various time points up to 7 dpi, as indicated.

**Protein extraction and purification**

Total proteins were extracted from infiltrated *N. benthamiana* leaves. Leaf tissue was grinded in liquid nitrogen and extracted in pre-chilled lysis buffer [50 mM Tris-HCl; 300 mM NaCl; 0.5% Triton X-100; 10 mM PMSF] at a ratio of 1:5 (w/v). The mixture was centrifuged to recover the supernatant. For affinity chromatography, a Ni-NTA 6FF gravity flow column (SANGON, China) was used to purify the extracted total soluble protein. The column was equilibrated by the equilibrium solution (50 mM Tris-HCl; 300 mM NaCl; 10 mM imidazole, pH=8.0). The soluble protein was filtered by 0.45 μM filter (Millipore, USA) before loading onto the equilibrated column by gravity flow. After loading, the Ni-NTA column was washed by wash buffer (50 mM Tris-HCl; 300 mM NaCl; 40 mM imidazole, pH=8.0) to remove non-specifically adsorbed impurities. The sample was eluted in 5 steps with elution buffer (50 mM Tris-HCl; 300 mM NaCl; 300 mM imidazole, pH=8.0) and was collected in Eppendorf tubes. Fractions from the different steps of the purification protocol were analyzed by SDS-PAGE and Western blot. The elution fraction containing the purified rhLHPP was concentrated by a Amicon Ultra 10KDa filter (Merck Millipore, USA). For purified rhLHPP mass estimation, fraction containing rhLHPP were compared with a dilution series of standard bovine serum albumin (Solarbio, China) by SDS-PAGE Coomassie Blue staining and densitometry analysis. Total soluble rhLHPP in the supernatant were determined by western blot and densitometry analysis, too. The band intensities were analyzed using Image J software **(**https://imagej.net/ij/index.html**).**

**Western blot analysis**

Proteins extracted or purified from previous sections were loaded in 10 % polyacrylamide gel (Shanghai Epizyme Biomedical Technology Co., Ltd, China) and electrophoresed for protein separation. After electrophoresis, gels were transferred to a 0.45 μm Polyvinylidene difluoride (PVDF) membrane (Beyotime, China) for antibody detection. His- tagged rhLHPP was detected using a primary anti-his mouse monoclonal antibody (TransGen Biotech, China). A plant specific primary anti-actin mouse monoclonal antibody (ABclonal, China) was used to detect the housekeeping protein β-actin. As secondary antibody, a horseradish peroxidase (HRP)-conjugated goat anti-mouse IgG antibody (TransGen Biotech, China) was used. The bound antibody was detected by chemiluminescence using ECL detection reagent (Thermo Scientific, USA) in accordance with the munufacturer’s procedures.

**Histochemical detection of reactive oxygen species**

Hydrogen peroxide accumulation was visualized using 3,3’-diaminobenzidine (DAB) staining (Solarbio, China). At 3 dpi, *N. benthamiana* leaves were harvested and immersed in freshly prepared DAB staining solution. Vacuum infiltration was performed at -0.1 MPa for 30 min to ensure uniform penetration of the staining solution. Subsequently, samples were incubated in darkness at room temperature for 12 h until distinct brown polymerization products developed in H_2_O_2_-rich regions, while unstained areas appeared light yellow or colorless. Following staining, leaves were carefully rinsed 3-5 times with distilled deionized water to remove excess DAB and blotted dry on absorbent paper. Decolorization was achieved by incubating samples in 95 % ethanol at 65 ℃ for 2-3 h, with ethanol changes as needed when the solution became chlorophyll-saturated. The process was continued until leaf backgrounds were completely destained, after which samples were photographed under consistent lighting conditions. Superoxide anion accumulation was detected using nitroblue tetrazaolium (NBT) staining (Solarbio, China) following an analogous protocol to DAB staining, with modifications for NBT-specific visualization of blue formazan precipitates.

**RNA isolation and quantitative PCR analysis**

Total RNA was isolated from *N. benthamiana* leaves using a plant Total RNA Rapid Extraction Kit (Biomed, China) following the manufacturer’s protocol. RNA integrity was verified by gel electrophoresis, and purity was assessed spectrophotometrically (Nanodrop One C, Thermo Scientific, USA) with acceptable A260/280 ratios between 1.8-2.0. The cDNA was synthesized from the total RNA using a PrimeScript™ RT reagent kit (Takara, Japan) with oligo (dT) primers. Quantitative real-time PCR was performed in triplicate using a LightCycler^®^ 480 Instrument II (Roche, Switzerland) with 96-well plates. Gene expression levels were normalized to the endogenous reference gene β-Actin and calculated using the comparative 2^-ΔΔCt method. All primer pairs were validated for amplification efficiency (90-110 %) and specificity by melt curve analysis.

**Virus-induced gene silencing assay**

*Agrobacterium tumefaciens* GV3101 strains harboring either pTRV1, pTRV2-PDS, or pTRV2-Rboh constructs were cultured in YEB media supplemented with appropriate antibiotics until reaching the mid-log phase (OD_600_=0.8). For plant infiltration, pTRV1 suspensions were mixed in a 1:1 ratio (v/v) with either pTRV2-PDS or pTRV2-Rboh suspensions. The mixed bacterial cultures were incubated at room temperature for 2 h in infiltration buffer. Subsequently, suspensions were infiltrated into leaves of 4-week-old *N. benthamiana* plants with syringes. When photobleaching in pTRV2-PDS-infiltrated plants was observed and upon confirmation of successful *Rboh* silencing by quantitative RT-PCR, *Agrobacterium* strains expressing rhLHPP were infiltrated into the silenced leaf areas for subsequent functional characterization.

**Confocal microscopy analysis**

*Agrobacterium*-infiltrated *N. benthamiana* leaves were examined on 3 dpi using a Leica STED super-resolution microscope (Leica Microsystems, Wetzlar, Germany). Epidermal cell observations were performed on the abaxial leaf surface with a 40× water immersion objective. Dual-channel imaging was performed simultaneously to assess protein colocalization. All images were acquired using LAS X software (Leica Microsystems).

**Bioinformatic analysis**

The predicted NbRboh protein sequence was aligned with its homologous sequences from *Arabidopsis thaliana* and relevant database entries, and phylogenetic analyses were subsequently performed with MEGA 11.0. The evolutionary analyses were conducted by using the Maximum Likelihood method based on the Jones-Taylor-Thornton (JTT) matrix-based model with bootstrap of 1000 replicates. All trees were drawn to scale; branch lengths and the number of substitutions per site were indicated in the corresponding units.

**Statistics analysis**

Statistical analyses were conducted using GraphPad Prism version 10.0. Data were analyzed using one-way ANOVA followed by Tukey’s multiple-comparison test (n=3). Group labels were assigned according to the compact letter display method, whereby groups denoted by different letters were considered to differ significantly.
